# Expression and Clinical Significance of the m6A RNA-Binding Proteins *YTHDF2* in Peripheral Blood Mononuclear Cells From New-Onset Ankylosing Spondylitis

**DOI:** 10.3389/fmed.2022.922219

**Published:** 2022-06-09

**Authors:** Qing Luo, Yongqin Guo, Qiuyun Xiao, Biqi Fu, Lu Zhang, Yang Guo, Zikun Huang, Junming Li

**Affiliations:** ^1^Department of Clinical Laboratory, The First Affiliated Hospital of Nanchang University, Nanchang, China; ^2^Medical College, Nanchang University, Nanchang, China; ^3^Department of Rheumatology, The First Affiliated Hospital of Nanchang University, Nanchang, China

**Keywords:** N6-methyladenosine, *YTHDF2*, ankylosing spondylitis, diagnosis, predictive model

## Abstract

This study has focused on determining the association of m6A methyltransferase [methyltransferase-like 3 (METTL3), methyltransferase-like 14 (METTL14), and Wilms tumor 1-associating protein (WTAP)], demethylase [fat mass and obesity-associated protein (FTO) and alkylation repair homolog protein 5 (ALKBH5)], RNA-binding proteins [YT521-B homology domains 2 (YTHDF2)], and ankylosing spondylitis (AS). A total of 154 specimens, containing 79 patients with new-onset AS and 75 healthy controls (HCs), participated in the study. The mRNA expressions of these m6A methyltransferase, demethylase, and RNA-binding protein in peripheral blood mononuclear cells (PBMCs) were detected by quantitative real-time PCR (qRT-PCR). The data showed that the mRNA expressions of *YTHDF2* and *ALKBH5* in PBMC from patients with new-onset AS were significantly decreased, and there was a positive correlation between RNA-binding proteins (*YTHDF2*) and demethylase (*ALKBH5*) in patients with new-onset AS. Logistic regression analysis demonstrated that the expression of *YTHDF2* mRNA in PBMC is a risk factor of AS. Receiver operating characteristic (ROC) analysis of the area under the curve (AUC) for mRNA *YTHDF2* in new-onset AS and HC was 0.692, with a cutoff value of <0.8724, a sensitivity of 67%, and a specificity of 63%. Moreover, we constructed a novel predictive model based on a combination of mRNA *YTHDF2* and systemic immune-inflammation index (SII) for AS diagnosis (AUC = 0.865, sensitivity = 79.45%, specificity = 84.00%), and the predictive model correlated with the activity and severity of AS. This study indicates that the mRNA expression of *YTHDF2* in PBMC may be involved in AS pathogenesis and a predictive model based on a combination of mRNA *YTHDF2* and SII acts as a marker for diagnosis and progression of diseases.

## Introduction

Ankylosing spondylitis (AS) is a type of rheumatic disease that leads to general inflammation and a debilitating form of arthritis involving the axial skeleton, with a global prevalence of about 0.1–0.5%, and often attack young and middle-aged men (1). Although the AS pathogenesis is mainly involved in the combined effects of environmental, infection, genetic, and other factors, the concrete pathogenesis remains unclear. Due to the limited understanding of disease pathogenesis, there is still a lack of specific diagnostic and therapeutic targets for AS (2), which usually cause a delay in onset diagnosis for AS and cannot get effective therapy in time (3).

N6-methyladenosine (m6A) modification is the most abundant internal modification which mainly occurs in mRNA and in most organisms (4). m6A modification regulates mRNA metabolism and is widely involved in multiple biological processes (5, 6). The m6A regulators primarily include methyltransferase that creates m6A mark, demethylase that erases m6A, and m6A RNA-binding proteins. It is well-known that methyltransferase-like 3 (METTL3), methyltransferase-like 14 (METTL14), and Wilms tumor 1-associating protein (WTAP) are classical m6A methyltransferases (7, 8). It has been shown that fat mass and obesity-associated protein (FTO) and alkylation repair homolog protein 5 (ALKBH5) are demethylases involving in m6A methylation reversion (9, 10). In addition, YT521-B homology domains 2 (YTHDF2) is an RNA-binding protein, which can recognize and decode m6A modification and finally modulate mRNA fate (11). Discrepant expression of the above m6A regulators results in abnormal expression of specific genes, which ultimately facilitates disease progression (12–17). Moreover, m6A modification and differential expression of key m6A regulators could use as diagnostic, prognostic, and therapeutic targets for many diseases (18–20). Moreover, Xie et al. demonstrated that METTL14-dependent m6A modification of ELMO1 contributes to the directional migration of mesenchymal stem cell (MSC) in AS (21). Although the m6A modification can affect the function of MSC in AS, the feature of m6A modification in peripheral blood mononuclear cells (PBMCs) in human AS is still unclear.

For the first time, we demonstrated that the mRNA levels of *YTHDF2* and *ALKBH5* in PBMC from patients with new-onset AS were significantly lower than that in healthy controls (HCs), and decreased *YTHDF2* was a risk factor for new-onset AS. Further research showed that *YTHDF2* exhibited a better predictive value for patients with new-onset AS, and the predictive model based on *YTHDF2* and systemic immune-inflammation index (SII) had an improvement value with the area under the curve (AUC) = 0.865. Moreover, the predictive model correlated with the activity and severity of AS. In total, YTHDF2 was proved to involve in the pathogenesis and be an independent predictive biomarker of new-onset AS.

## Materials and Methods

### Patient Variables and Controls

Potential patients for this study were consecutively enrolled between September 2019 and June 2021 at The First Affiliated Hospital of Nanchang University. The patients with AS ultimately included herein all fulfilled the modified New York 1984 criteria for AS; those patients with AS with other autoimmune, inflammatory, or hormonal diseases, cancers, or mental disorders were excluded. All AS cases were new-onset, and the subjects had not yet used immunosuppressive agents or corticosteroids prior to blood sample collection. In the beginning of the study, a total of 79 subjects with AS were evaluated. Healthy age-matched control (HC; *n* = 75) subjects without autoimmune diseases and free of other inflammatory conditions were randomly recruited at the same hospital at the same time. The research protocol complied with the principles outlined in the Declaration of Helsinki and was approved by the Institutional Ethics Committee of our hospital. All subjects who participated in this research provided signed informed consent before tests.

### Isolation of Peripheral Blood Mononuclear Cell Samples and RNA Extraction

To isolate PBMC for analyses, fasting blood (≈ 3 ml) was collected from the elbow vein into EDTA-coated and the PBMC was then isolated using previously reported protocols (22). After the determination of cell concentrations in each isolated sample, RNA was isolated from 2 × 10^6^ PBMC/patient using a 0.75 ml TRIzol reagent (Invitrogen Bio, Waltham, MA, United States), according to the manufacturer’s protocols. After incubating the homogenized samples (2 × 10^6^ PBMC + 0.75 ml TRIzol reagent) for 5 min, 0.2 ml of chloroform was used for phase separation (by shaking, incubation, centrifugation, and transferring the aqueous phase) 0.5 ml of isopropyl alcohol was used for RNA precipitate (by incubation, centrifugation, and forming a gel-like pellet), at least 1 ml of 75% ethanol was added for RNA wash (by mixing and centrifugation), and then the RNA was redissolved in RNase-free water. The concentration and purity of each total RNA isolate were determined (using A260/A280 and A260/A230 ratios) using a NanoDrop ND-1000 spectrophotometer (Invitrogen Bio, Waltham, MA, United States).

### Quantitative Real-Time PCR Analysis

For PCR analyses, 1 μg total RNA/isolate was used to synthesize cDNA by reverse transcription using a PrimeScript RT reagent kit (Takara Bio, Tokyo, Japan) following the manufacturer’s protocols. The product, in turn, was used as a template for PCR in an ABI 7500 Real-Time PCR System (Invitrogen Bio, Waltham, MA, United States) that employed the SYBR Green detection method (Takara Bio, Tokyo, Japan). The primers for *YTHDF2*, *ALKBH5*, *FTO*, *METTL3*, *METTL14*, *WTAP*, and *GAPDH* (housekeeping gene) are presented in Table 1. All qRT-PCR experiments were performed in triplicate. The result of the above gene was derived using the 2^–ΔΔCt^ method (23).

**TABLE 1:** The qRT-PCR sequences.

Gene	Sequence (F: 5′-3′)	Sequence (R: 5′-3′)
*YTHDF2*	GGCAGCACTGAAGTTGGG	CTATTGGAAGCCACGATGTTA
*ALKBH5*	CCCGAGGGCTTCGTCAACA	CGACACCCGAATAGGCTTGA
*FTO*	TGGGTTCATCCTACAACGG	CCTCTTCAGGGCCTTCAC
*WTAP*	GGCGAAGTGTCGAATGCT	CCAACTGCTGGCGTGTCT
*METTL3*	AAGCTGCACTTCAGACGAAT	GGAATCACCTCCGACACTC
*METTL14*	AGAAACTTGCAGGGCTTCCT	TCTTCTTCATATGGCAAATTTTCTT
*GAPDH*	TGCACCACCAACTGCTTAGC	GGCATGGACTGTGGTCATGAG

*ALKBH5, alkylation repair homolog protein 5; FTO, fat mass and obesity-associated protein; GAPDH, glyceraldehyde-3-phosphate dehydrogenase; METTL3, methyltransferase-like 3; METTL14, methyltransferase like 14; qRT-PCR, quantitative real-time PCR; WTAP, Wilms tumor 1-associating protein; YTHDF2, YT521-B homology domains 2.*

### Clinical Assessments and Laboratory Indexes

Over the course of the study, all subjects had clinical and laboratorial information collected by trained staff. The disease activity of AS was evaluated by the Bath AS Functional Index (BASFI), the Bath AS Disease Activity Index (BASDAI), and the AS Disease Activity Score-C reaction protein (ASDAS-CRP) (24, 25). Major histocompatibility complex-class I-B27 (HLA-B27) was measured by the abovementioned PCR. Serum levels of CRP were evaluated using an IMMUNE 800 system (Beckman Coulter, San Jose, CA, United States). Erythrocyte sedimentation rate (ESR) was detected using an Automatic ESR analyzer TEST1 system (ALIFAX Bio, Udine, Italy). Blood routine parameters, including the white blood cell (WBC) and red blood cell (RBC) counts, hemoglobin (HGB), hematocrit (HCT), platelet count (PLT), mean platelet volume (MPV), plateletcrit (PCT), platelet distribution width (PDW), lymphocyte counts (L) and percentages (L%), monocyte counts (M) and percentages (M%), neutrophil counts (N), and percentages (N%), were examined by a Sysmex XE-2100 analyzer (Sysmex Bio, Kobe, Japan). In addition, novel inflammatory markers, including lymphocyte-to-monocyte ratio (LMR), platelet-to-lymphocyte ratio (PLR), neutrophil-to-lymphocyte ratio (NLR), derived neutrophil-lymphocyte ratio (dNLR), platelet-to-monocyte ratio (PMR), monocyte-to-neutrophil ratio (MNR), monocyte-to-lymphocyte ratio (MLR), platelet-to-neutrophil ratio (PNR), and SII were calculated according to blood routine parameters.

### Statistical Analysis

All data are expressed in terms of means ± standard error. For the analyses, an unpaired *t* test or Mann–Whitney *U* test was performed to identify statistical differences among groups according to the normality. The Spearman method and multivariate regression analysis (logistic regression) were used for correlation analysis and risk factors analysis, respectively. The SPSS version 17.0 (SPSS Bio, Chicago, IL, United States) and Prism version 5.0 (GraphPad Bio, San Diego, CA, United States) software were used for data analysis. *P*-value ≤ 0.05 was considered statistically significant.

## Results

### Characteristics of the Study Population

This study enrolled a total of 258 participants, i.e., 79 patients with new-onset AS and 75 HCs. Population characteristics are detailed in Table 2. The new-onset AS group and HC group were matched for age and gender for all analyses. Moreover, there were significant differences between the two groups in some blood routine parameters and novel inflammatory markers, including LMR, PLR, NLR, dNLR, PMR, MNR, PNR, and SII (Table 2).

**TABLE 2:** Clinical details of subjects with patients with new-onset AS and HC.

Clinical characteristic	AS (*n* = 79)	HC (*n* = 75)	*P* value
Sex (male/female)	55/24	55/20	0.6100
Age (years)	32.25 ± 10.46	32.99 ± 9.16	0.6447
BASFI	3.95 ± 1.42		
BASDAI	4.32 ± 1.66		
ASDAS-CRP	2.83 ± 1.14		
HLA-B27 positive (out of 63 patients tested)	53		
ESR (mm/h)	21.35 ± 21.44		
CRP (mg/l)	18.02 ± 28.48		
WBC (10^9^/l)	7.51 ± 2.53^a^	6.39 ± 1.22	0.0002
RBC (10^12^/l)	4.85 ± 0.68^a^	5.04 ± 0.43	0.0330
HGB (g/l)	138.89 ± 17.03^a^	150.11 ± 21.35	0.0005
HCT (l/l)	0.43 ± 0.05^a^	0.45 ± 0.04	0.0007
PLT (10^9^/l)	290.45 ± 84.91^a^	237.63 ± 39.98	<0.0001
MPV (fl)	9.76 ± 1.10^a^	10.58 ± 0.79	<0.0001
PCT (%)	0.28 ± 0.07^a^	0.25 ± 0.039	0.0178
PDW (fl)	14.65 ± 2.41^a^	12.74 ± 1.64	<0.0001
L (10^9^/l)	1.87 ± 0.51^a^	2.18 ± 0.47	0.0002
L (%)	26.06 ± 6.42^a^	34.7 ± 7.16	<0.0001
M (10^9^/l)	0.44 ± 0.14	0.45 ± 0.14	0.6161
M (%)	6.03 ± 1.58^a^	7.07 ± 1.68	0.0002
N (10^9^/l)	5.03 ± 2.35^a^	3.59 ± 0.98	<0.0001
N (%)	65.71 ± 7.59^a^	55.68 ± 7.31	<0.0001
PLR	165.65 ± 66.75^a^	114.27 ± 35.92	<0.0001
NLR	2.85 ± 1.54^a^	1.76 ± 0.90	<0.0001
dNLR	2.11 ± 1.00^a^	1.33 ± 0.48	<0.0001
LMR	4.55 ± 1.56^a^	5.21 ± 1.67	0.0143
PMR	692.12 ± 214.39^a^	572.18 ± 190.82	0.0004
MNR	0.09 ± 0.03^a^	0.13 ± 0.04	<0.0001
PNR	63.12 ± 22.48^a^	70.81 ± 21.55	0.0347
SII	874.41 ± 781.45^a^	412.51 ± 203.93	<0.0001

*^a^<0.05 AS compared to HC.*

*AS, ankylosing spondylitis; ASDAS, AS Disease Activity Score; BASDAI, Bath AS Disease Activity Index; BASFI, Bath AS Functional Index; CRP, C reaction protein; dNLR, derived neutrophil-lymphocyte; ESR, erythrocyte sedimentation rate; HC, healthy control; HCT, hematocrit; HGB, hemoglobin; HLA-B27, major histocompatibility complex-class I-B27; L, lymphocyte counts; L%, lymphocyte percentages; LMR, lymphocyte-to-monocyte; M, monocyte counts; M%, monocyte percentages; MNR, monocyte-to-neutrophil; MPV, mean platelet volume; N, neutrophils counts; N%, neutrophil percentages; NLR, neutrophil-to-lymphocyte; PCT, plateletcrit; PDW, platelet distribution width; PLR, platelet-to-lymphocyte; PLT, platelet count; PMR, platelet-to-monocyte; PNR, platelet-to-neutrophil; RBC, red blood cell counts; SII, systemic immune-inflammation index; WBC, white blood cell counts.*

### The Expression of mRNA *YTHDF2, ALKBH5, FTO, WTAP, METTL3*, and *METTL14* in Peripheral Blood Mononuclear Cell From Patients With New-Onset Ankylosing Spondylitis and Healthy Control

**FIGURE 1:**
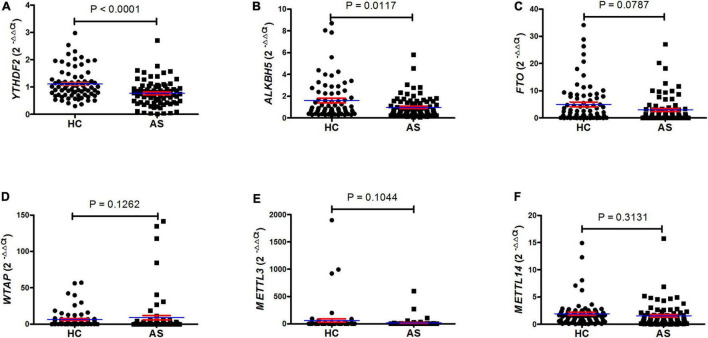
QRT-PCR showed that patients with AS have a lower level of mRNA *YTHDF2* and *ALKBH5* in PBMC. **(A)** The level of mRNA *YTHDF2* between patients with AS and HC (unpaired *t* test). **(B)** The level of mRNA *ALKBH5* between patients with AS and HC (Mann–Whitney *U* test). **(C)** The level of mRNA *FTO* between patients with AS and HC (Mann–Whitney *U* test). **(D)** The level of mRNA *WTAP* between patients with AS and HC (Mann–Whitney *U* test). **(E)** The level of mRNA *METTL3* between patients with AS and HC (Mann–Whitney *U* test). **(F)** The level of mRNA *METTL14* between patients with AS and HC (Student’s *t* test). ALKBH5, alkylation repair homolog protein 5; AS, ankylosing spondylitis; FTO, fat mass and obesity-associated protein; HC, healthy control; METTL3, methyltransferase-like 3; METTL14, methyltransferase like 14; qRT-PCR, quantitative real-time PCR; WTAP, Wilms tumor 1-associating protein; YTHDF2, YT521-B homology domains 2. Blue and red lines indicates mean and standard error, respectively.

To examine the mRNA expression of m6A RNA-binding proteins (*YTHDF2*), demethylases (*ALKBH5* and *FTO*), and methyltransferases (*WTAP*, *METTL3*, and *METTL14*) in PBMC in patients with new-onset AS and HC, we adopted qRT-PCR to explore these genes expression in PBMC. The expression of mRNA *YTHDF2* and *ALKBH5* in PBMCs was significantly decreased in the patient group with new-onset AS compared to the HC group (*P* < 0.0001, Figure 1A; *P* = 0.0117, Figure 1B), whereas the expression of mRNA FTO, WTAP, METTL3, and METTL14 was unchanged (all *P* > 0.0500, Figures 1C–F).

### Relationship Between Clinical Variables and Expression Levels of *YTHDF2* and *ALKBH5* in Peripheral Blood Mononuclear Cells From Patients With New-Onset Ankylosing Spondylitis

**FIGURE 2:**
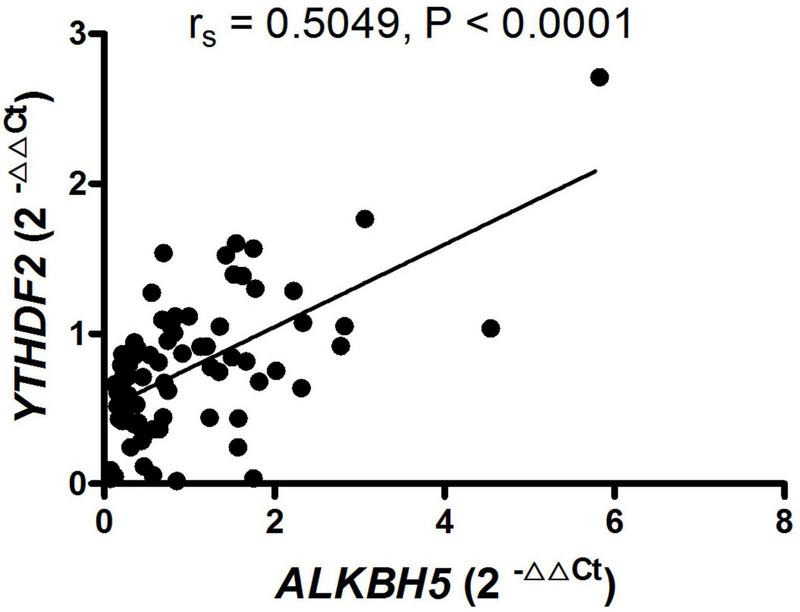
RNA-binding proteins (*YTHDF2*) positively correlated with demethylase (*ALKBH5*) in patients with new-onset AS. ALKBH5, alkylation repair homolog protein 5; AS, ankylosing spondylitis; YTHDF2, YT521-B homology domains 2.

To determine whether the expression of mRNA *YTHDF2* and *ALKBH5* in PBMC from patients with new-onset AS was a relevant indicator for the activity and severity of AS, we used the Spearman correlation test to determine the relationship between the clinical variables of AS and the expression of mRNA *YTHDF2* and *ALKBH5*. However, the expression of mRNA *YTHDF2* and *ALKBH5* in PBMC from patients with new-onset AS did not correlate with clinical variables that indicates the activity and severity of the disease, including BASFI, BASDAI, ASDAS-CRP, ESR, CRP, WBC, RBC, HGB, HCT, PLT, MPV, PCT, PDW, L, L%, M, M%, N, N%, PLR, NLR, dNLR, LMR, PMR, PNR, MNR, SII, and HLA-B27 (data not shown). In addition, the expression of mRNA *YTHDF2* correlated with the expression of mRNA *ALKBH5* in patients with new-onset AS (*r*_*s*_ = 0.5049, *P* < 0.0001, Figure 2).

### The Expression of mRNA *YTHDF2* in Peripheral Blood Mononuclear Cell Was a Risk Factor for New-Onset Ankylosing Spondylitis

The above results demonstrated that the expression of *YTHDF2* and *ALKBH5* in PBMC from patients with new-onset AS was obviously downregulated, and did not correlate with the activity and severity of AS. Thus, in six m6A regulators (i.e., *YTHDF2*, *ALKBH5*, *FTO*, *WTAP*, *METTL3*, and *METTL14*), we selected decreased expression of mRNA *YTHDF2* and *ALKBH5* to investigate whether the two decreased m6A regulators were risk factors for new-onset AS by the “enter method” of logistic regression. As shown in Table 3, the equations about the expression of mRNA *YTHDF2* and *ALKBH5* were obtained, *Y* = 1.386−1.456 × *YTHDF2*−0.006 × *ALKBH5*. In addition, the logistic regression analysis indicated that the downregulated expression of mRNA *YTHDF2* in PBMC was a risk factor for patients with new-onset AS (*P* = 0.0030), whereas the downregulated expression of mRNA *ALKBH5* in PBMC was not a risk factor (*P* = 0.9700).

**TABLE 3:** The mRNA expression of *YTHDF2* and *ALKBH5* in equation.

		*B*	*SE*	Wald	*Df*	*P*	Exp(*B*)
AS vs. HC	YTHDF2	−1.456	0.484	9.045	1	0.0030	0.233
	ALKBH5	−0.006	0.165	0.001	1	0.9700	0.994
	Constant	1.386	0.395	12.311	1	<0.0001	3.998

*ALKBH5, alkylation repair homolog protein 5; AS, ankylosing spondylitis; HC, healthy control; SE, standard error; YTHDF2, YT521-B homology domains 2.*

### Using *YTHDF2* and Routine Laboratory Indicators for Predicting Patients With New-Onset Ankylosing Spondylitis

**FIGURE 3:**
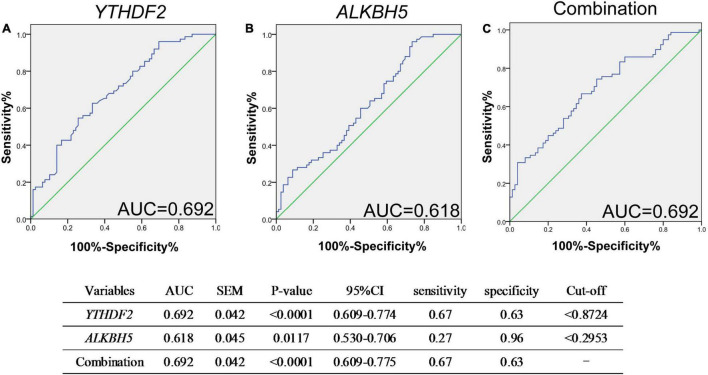
ROC analysis showed that mRNA *YTHDF2* has a better predictive value for AS. **(A)** ROC analysis of mRNA *YTHDF2.* **(B)** ROC analysis of mRNA *ALKBH5.* **(C)** ROC analysis of combination of mRNA *YTHDF2* and *ALKBH5.* ALKBH5, alkylation repair homolog protein 5; AS, ankylosing spondylitis; ROC, receiver operating characteristic; YTHDF2, YT521-B homology domains 2. Blue and green lines indicates ROC and line of identity, respectively.

**FIGURE 4:**
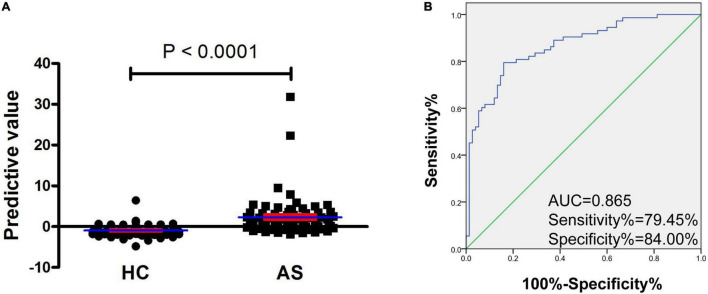
Increased predictive model based on mRNA *YTHDF2* and SII has an improvement value for AS. **(A)** The predictive value between patients with AS and HC. Blue and red lines indicates mean and standard error, respectively. **(B)** ROC analysis of predictive model. Blue and green lines indicates ROC and line of identity, respectively. AS, ankylosing spondylitis; HC, healthy control; ROC, receiver operating characteristic; SII, systemic immune-inflammation index; YTHDF2, YT521-B homology domains 2.

Next, we performed a receiver operating characteristic (ROC) curve to explore the potential predictive value of mRNA *YTHDF2* and *ALKBH5* in PBMC for new-onset AS, and the values showed that the AUC of mRNA *YTHDF2* for distinguishing new-onset AS from HC was 0.692, with a cutoff value of <0.8724, a sensitivity of 67%, and a specificity of 63% (Figure 3A), the AUC of mRNA *ALKBH5* was 0.618, with a cutoff value of <0.2953, a sensitivity of 27%, and a specificity of 96% (Figure 4B), and the AUC of the combination of mRNA *ALKBH5* and *YTHDF2* for distinguishing new-onset AS from HC was 0.692, with a sensitivity of 67% and a specificity of 63% (Figure 3C). The combination of *ALKBH5* and *YTHDF2* (0.692) had no improvement in predicting patients with new-onset AS when compared with the sole use of *YTHDF2* (0.692).

As presented in Table 4, five routine laboratory indicators, including L%, N%, NLR, dNLR, and SII, had the potential in predicting new-onset AS from HC, with AUC > 0.8. To determine the predictive model based on the combination of mRNA *YTHDF2* and routine laboratory indicators for distinguishing patients with new-onset AS from HC, and with a view to the less number of subjects, we selected mRNA *YTHDF2* and all routine laboratory indicators with AUC > 0.8 for further univariable and multivariable analyses. Based on the multivariate analysis, *YTHDF2* and SII were selected as variables for the predictive model (Table 5). Based on regression coefficients, we built a model to predict patients with new-onset AS from HC as follows: *P* = 0.006 × SII−1.372 × *YTHDF2*−1.877 where P is the predictive value. The value of each subject was reckoned, and a greater value would predict a higher probability for new-onset AS (Figure 4A).

**TABLE 4:** The performance of routine indicators.

Item	AUC	Sensitivity (%)	Specificity (%)
WBC (10^9^/l)	0.678	64.86	72.00
RBC (10^12^/l)	0.601	51.35	70.67
HGB (g/l)	0.722	71.62	65.33
HCT (l/l)	0.662	54.05	76.00
PLT (10^9^/l)	0.694	45.95	93.33
MPV (fl)	0.750	58.11	86.49
PCT (%)	0.613	47.3	79.73
PDW (fl)	0.765	68.92	91.89
L (10^9^/l)	0.682	58.11	78.67
L (%)	0.821	74.32	76.00
M (%)	0.700	75.68	65.33
N (10^9^/l)	0.766	72.97	74.67
N (%)	0.842	75.68	78.67
PLR	0.752	58.11	88.00
NLR	0.819	72.97	76.00
dNLR	0.835	75.68	78.67
LMR	0.621	68.92	60.00
PMR	0.670	51.35	77.33
MNR	0.795	70.27	80.00
PNR	0.613	54.05	69.33
SII	0.832	62.16	96.00

*AUC, area under the curve; dNLR, derived neutrophil-lymphocyte; HCT, hematocrit; HGB, hemoglobin; L, lymphocyte counts; L%, lymphocyte percentages; LMR, lymphocyte-to-monocyte; M, monocyte counts; MNR, monocyte-to-neutrophil; MPV, mean platelet volume; N, neutrophils counts; N%, neutrophil percentages; NLR, neutrophil-to-lymphocyte; PCT, plateletcrit; PDW, platelet distribution width; PLR, platelet-to-lymphocyte; PLT, platelet count; PMR, platelet-to-monocyte; PNR, platelet-to-neutrophil; RBC, red blood cell counts; SII, systemic immune-inflammation index; WBC, white blood cell counts.*

**TABLE 5:** Univariable and multivariable analysis of risk factors correlated with new-onset AS.

	Univariate analysis			Multivariate analysis		
	OR	95% CI	*P*-value	OR	95% CI	*P*-value
*YTHDF2*	−1.467	0.107–0.498	<0.0001	−1.174	0.123–0.775	0.0120
L%	−0.200	0.764–0.878	<0.0001			
N%	0.208	1.144–1.325	<0.0001			
NLR	1.534	2.476–8.687	<0.0001			
dNLR	2.707	5.328–42.149	<0.0001			
SII	0.006	1.004–1.008	<0.0001	0.006	1.002–1.010	0.0030

*CI, confidence interval; dNLR, derived neutrophil-lymphocyte; L%, lymphocyte percentages; N%, neutrophil percentages; NLR, neutrophil-to-lymphocyte; OR, odds ratio; SII, systemic immune-inflammation index; YTHDF2, YT521-B homology domains 2.*

The predictive model based on a combination of mRNA *YTHDF2* and SII performed best in distinguishing patients with new-onset AS from HC with AUC of 0.865 (95% CI, 0.807–0.923) (Figure 4B), which was superior to mRNA *YTHDF2* (Figure 3), SII, and other routine laboratory indicators (Table 1). When the cutoff value of the predictive model was −0.099, the sensitivity was 79.45% and the specificity was 84.00%.

**FIGURE 5:**
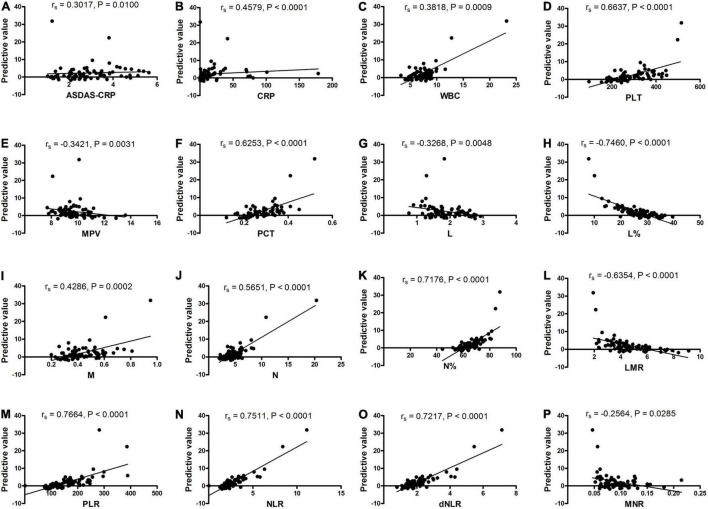
The predictive model correlated with the activity and severity of AS. **(A)** The predictive model correlated with ASDAS-CRP. **(B)** The predictive model correlated with CRP. **(C)** The predictive model correlated with WBC. **(D)** The predictive model correlated with PLT. **(E)** The predictive model correlated with MPV. **(F)** The predictive model correlated with PCT. **(G)** The predictive model correlated with L. **(H)** The predictive model correlated with L%. **(I)** The predictive model correlated with M. **(J)** The predictive model correlated with N. **(K)** The predictive model correlated with N%. **(L)** The predictive model correlated with LMR. **(M)** The predictive model correlated with PLR. **(N)** The predictive model correlated with NLR. **(O)** The predictive model correlated with dNLR. **(P)** The predictive model correlated with MNR. AS, ankylosing spondylitis; ASDAS, AS Disease Activity Score; CRP, C reaction protein; dNLR, derived neutrophil-lymphocyte; L, lymphocyte counts; L%, lymphocyte percentages; LMR, lymphocyte-to-monocyte; M, monocyte counts; MNR, monocyte-to-neutrophil; MPV, mean platelet volume; N, neutrophils counts; N%, neutrophil percentages; NLR, neutrophil-to-lymphocyte; PCT, plateletcrit; PLR, platelet-to-lymphocyte; PLT, platelet count; WBC, white blood cell counts.

Moreover, the correlation between the predictive model and the severity of new-onset AS was explored and the results (Figure 5) showed that the predictive model based on a combination of mRNA *YTHDF2* and SII correlated with clinical severity indicators of AS, including ASDAS-CRP (*r*_*s*_ = 0.3017, *P* = 0.0100), CRP (*r*_*s*_ = 0.4579, *P* < 0.0001), WBC (*r*_*s*_ = 0.3818, *P* = 0.0009), PLT (*r*_*s*_ = 0.6637, *P* < 0.0001), MPV (*r*_*s*_ = −0.3421, *P* = 0.0031), PCT (*r*_*s*_ = 0.6253, *P* < 0.0001), L (*r*_*s*_ = −0.3269, *P* = 0.0048), L% (*r*_*s*_ = −0.7460, *P* < 0.0001), M (*r*_*s*_ = 0.4286, *P* = 0.0002), N (*r*_*s*_ = 0.5651, *P* < 0.0001), N% (*r*_*s*_ = 0.7176, *P* < 0.0001), LMR (*r*_*s*_ = −0.6354, *P* < 0.0001), PLR (*r*_*s*_ = 0.7664, *P* < 0.0001), NLR (*r*_*s*_ = 0.7511, *P* < 0.0001), dNLR (*r*_*s*_ = 0.7217, *P* < 0.0001), and MNR (*r*_*s*_ = −0.2564, *P* = 0.0285).

## Discussion

m6A is the most abundant internal modification of mRNA in the majority of eukaryotes, and increasing studies focus on the effect of m6A methylation of RNA in inflammation and autoimmunity (26, 27). There are many key regulators that participated in m6A methylation, mainly including YTHDF2, ALKBH5, FTO, WTAP, METTL3, and METTL14. The current study first examined the mRNA expression of m6A regulators (*YTHDF2*, *ALKBH5*, *FTO*, *WTAP*, *METTL3*, and *METTL14*) in PBMC from patients with AS and healthy counterparts. The analyses indicated that the *YTHDF2* and *ALKBH5* mRNA expression in PBMC from patients with AS was significantly lower than in cells from the HC, whereas the mRNA expression of *FTO*, *WTAP*, *METTL3*, and *METTL14* was unchanged. Recently, Xie et al. explored the level of ALKBH5, FTO, WTAP, METTL3, and METTL14 in MSC with or without tumor necrosis factor alpha (TNF-α) stimulation from patients with AS and showed inconsistent results that only METTL14 was clearly downregulated in MSC from AS (21). The causes for the conflicting consequences may be due to discrepancies in cell type and TNF-α stimulation.

The evidence manifested that the mRNA level of PBMC m6A demethylases ALKBH5 positively correlated with that of RNA-binding protein YTHDF2 in patients with AS, which is consistent with the data from Yang et al. (28) and our previous study in systemic lupus erythematosus (SLE) (29). Given the factors that the cooperation among demethylases and RNA-binding protein establishes the m6A threshold value and disrupts the threshold value results in abnormal activation/expression of causal gene and contributes to disease occurrence and progression (30), the result in this study indicated that the depressed YTHDF2 and ALKBH5 may involve in AS pathogenesis.

At present, clinical characteristics, imageological changes, HLA-B27, and acute phase reactants (ESR and CRP) are the primary means to diagnose AS. However, many HLA-B27 (−) patients have early clinical characteristics and imaging changes are atypical, and acute phase reactants are not high, which brings enormous diagnostic challenges to us. Evidence from many studies has indicated that differential expression of key m6A moderators could use as prognostic and therapeutic targets for many diseases (18–20). Furthermore, HNRNPA2B1 and HNRNPC that are m6A RNA-binding proteins were demonstrated to serve as useful diagnostic biomarkers for endometriosis (20). The current study examined the potential predictive value of decreased mRNA expression of *YTHDF2* and *ALKBH5* for AS, and the results showed that the AUC for *YTHDF2* in AS and HC was 0.692, the AUC for ALKBH5 in AS and HC was 0.618, and the AUC for the combination of *ALKBH5* and *YTHDF2* in patients with AS and HC was 0.692, suggesting that the predictive value of *YTHDF2* was superior to *ALKBH5* and the predictive value of combination based on *ALKBH5* and *YTHDF2* had no improvement in predicting patients with AS when compared with *YTHDF2.*

Although our results demonstrated the expression of mRNA *YTHDF2*, *ALKBH5* was obviously decreased in patients with AS, and decreased expression of mRNA *YTHDF2* and *ALKBH5* had some value in predicting patients with AS, these results were inconsistent with the fact that the logistic regression analysis manifested the downregulated expression of mRNA *YTHDF2* in PBMC was a risk factor for patients with AS, whereas the downregulated expression of mRNA *ALKBH5* in PBMC was not. The reason for the conflicting results may be that *ALKBH5* is concomitantly decreased by *YTHDF2* and YTHDF2 may play a dominant role in AS pathogenesis. In addition, research has indicated that different m6A regulators can constitute a feedback loop with RNA stability factor to regulate the stability of target transcripts (30), and concomitant changes in the m6A regulator had been found in other autoimmune diseases (29, 31).

Recently, many studies manifested that the blood routine parameters and the rate between them may use as indicators for state, development, and prognosis of diseases (32, 33). AS is a common chronic rheumatic disease, and we systematically determined the relevance between new-onset AS and blood routine parameters (WBC, L, L%, N, N%, M, M%, PLT, MPV, PCT, PDW, RBC, HGB, and HCT) and the ratio between them (LMR, PLR, NLR, dNLR, PMR, MNR, PNR, and SII). In agreement with previous studies (34–36), there were significant differences between AS group and control group in WBC, RBC, HGB, HCT, PLT, MPV, PCT, PDW, L, L%, M%, N, N%, LMR, PLR, NLR, dNLR, PMR, MNR, PNR, and SII, and HGB, MPV, PDW, M%, N, PLR, and MNR had a predictive value for AS with AUC > 0.700, especially, L%, N%, NLR, dNLR, and SII had a better effect with AUC > 0.800. Albeit some studies have paid close attention to the AS-related risk factors, it is rare to model the relationship among different factors (35). As far as we know, this is the first study to set up a mathematical model based on m6A RNA-binding proteins and L%, N%, NLR, dNLR, and SII for predicting AS. To our surprise, *YTHDF2* and SII, but not L%, N%, NLR, and dNLR, were brought into the mathematical model, indicating *YTHDF2* and SII have a synergistic role in predicting AS. In addition, the predictive model based on *YTHDF2* and SII performed best in distinguishing patients with AS from HC with AUC of 0.865 (sensitivity = 79.45%, specificity = 84.00%), which was superior to YTHDF2, SII, and other routine laboratory indicators.

Moreover, many reports have explored the relevance between *YTHDF2*, *ALKBH5*, and clinical features of autoimmune disease. In rheumatoid arthritis (RA), our previous study has demonstrated that the expression of mRNA *YTHDF2* correlated with N%, L%, NLR, LMR, and RBC, whereas no correlation was found between *ALKBH5* and clinical features of RA (37). In addition, in SLE, the level of mRNA *ALKBH5* was associated with autoantibody production, WBC, ulceration, and rash, while no correlation was found between *YTHDF2* and clinical features of SLE (29). In this study, we also analyzed the association between level of mRNA *ALKBH5*, *YTHDF2*, and clinical features of AS, but we did not find any correlation. To our surprise, the predictive model based on *YTHDF2* and SII correlated with clinical severity indicators of AS, including ASDAS-CRP, CRP, WBC, PLT, MPV, PCT, L, L%, PLR, M, LMR, N, N%, NLR, dNLR, and MNR. These results indicated that the predictive model based on *YTHDF2* and SII could use as an indicator of disease activity and severity.

This study had some limitations. First, the samples were collected from only one institution, which may lead to a certain risk of bias. In addition, multicenter clinical studies with a larger sample size remain needed. Second, due to SII also obviously increases in many other inflammatory diseases besides AS, this study only used HC as the control group may result in the predictive model based on *YTHDF2* and SII will have a limited value in real clinical practice. Moreover, follow-up studies with other inflammatory diseases as disease control are needed. Third, the m6A modification expression and the mechanism of decreased YTHDF2 and ALKBH5 in AS pathogenesis should be explored in the future.

## Conclusion

The current study first measured the mRNA expression of m6A regulators in PBMC of AS and HC, and manifested that the mRNA expressions of *YTHDF2* and *ALKBH5* in PBMC were decreased in patients with AS. In addition, the logistic regression analysis demonstrated that the mRNA expression of *YTHDF2* in PBMC is a risk factor of AS. Moreover, mRNA *YTHDF2* and predictive model based on a combination of mRNA *YTHDF2* and SII may serve as a novel, non-invasive predictive biomarkers for AS, a predictive model, as well as act as indicators of disease severity and activity. The clinical studies with a larger sample size and other inflammatory diseases as disease control, and the precise molecular mechanisms underlying the functions of *YTHDF2* in AS still require further investigation.

## Data Availability Statement

The original contributions presented in this study are included in the article/supplementary material, further inquiries can be directed to the corresponding authors.

## Ethics Statement

The studies involving human participants complied with the principles outlined in the Declaration of Helsinki and were approved by the Institutional Ethics Committee of our Hospital. The patients/participants provided their written informed consent to participate in this study.

## Author Contributions

QL participated in designing the study, performed the statistical analyses, and drafted the manuscript. YoG carried out qRT-PCR analysis, data acquisition, performed the statistical analyses, and helped to revise the manuscript. QX carried out qRT-PCR analysis and drafted the manuscript. BF performed the statistical analyses and drafted the manuscript. LZ carried out the data acquisition and drafted the manuscript. YaG carried out data acquisition, performed the statistical analyses, and drafted the manuscript. ZH and JL conceived the study, participated in its design and coordination, and helped to draft the manuscript. All authors read and approved the final manuscript.

## Conflict of Interest

The authors declare that the research was conducted in the absence of any commercial or financial relationships that could be construed as a potential conflict of interest.

## Publisher’s Note

All claims expressed in this article are solely those of the authors and do not necessarily represent those of their affiliated organizations, or those of the publisher, the editors and the reviewers. Any product that may be evaluated in this article, or claim that may be made by its manufacturer, is not guaranteed or endorsed by the publisher.

## References

[1] Taurog JD Chhabra A Colbert RA. Ankylosing spondylitis and axial spondyloarthritis. *N Engl J Med.* (2016) 374:2563–74. 27355535 10.1056/NEJMra1406182

[2] Ward MM Deodhar A Gensler LS Dubreuil M Yu D Khan MA 2019 update of the American college of rheumatology/spondylitis association of America/spondyloarthritis research and treatment network recommendations for the treatment of ankylosing spondylitis and nonradiographic axial spondyloarthritis. *Arthritis Rheumatol.* (2019) 71:1599–613. 31436036 10.1002/art.41042 PMC6764882

[3] Maksymowych WP. Biomarkers for diagnosis of axial spondyloarthritis, disease activity, prognosis, and prediction of response to therapy. *Front Immunol.* (2019) 10:305. 10.3389/fimmu.2019.00305 30899255 PMC6416369

[4] Wang X Lu Z Gomez A Hon GC Yue Y Han D N6-methyladenosinedependent regulation of messenger RNA stability. *Nature.* (2014) 505:117–20. 24284625 10.1038/nature12730 PMC3877715

[5] Frye M Harada BT Behm M He C. RNA modifications modulate gene expression during development. *Science.* (2018) 361:1346–9. 10.1126/science.aau1646 30262497 PMC6436390

[6] Zaccara S Ries RJ Jaffrey SR. Reading, writing and erasing mRNA methylation. *Nat Rev Mol Cell Biol.* (2019) 20:608–24. 10.1038/s41580-019-0168-5 31520073

[7] Liu J Yue Y Han D Wang X Fu Y Zhang L METTL3-METTL14 complex mediates mammalian nuclear RNA N6-adenosine methylation. *Nat Chem Biol.* (2014) 10:93–5. 10.1038/nchembio.1432 24316715 PMC3911877

[8] Ping XL Sun BF Wang L Xiao W Yang X Wang WJ Mammalian WTAP is a regulatory subunit of the RNA N6-methyladenosine methyltransferase. *Cell Res.* (2014) 24:177–89. 10.1038/cr.2014.3 24407421 PMC3915904

[9] Jia G Fu Y Zhao X Dai Q Zheng G Yang Y N6-methyladenosine in nuclear RNA is a major substrate of the obesity-associated FTO. *Nat Chem Biol.* (2011) 7:885–7. 10.1038/nchembio.687 22002720 PMC3218240

[10] Zheng G Dahl JA Niu Y Fedorcsak P Huang CM Li CJ ALKBH5 is a mammalian RNA demethylase that impacts RNA metabolism and mouse fertility. *Mol Cell.* (2013) 49:18–29. 10.1016/j.molcel.2012.10.015 23177736 PMC3646334

[11] Maity A Das B. N6-methyladenosine modification in mRNA: machinery, function and implications for health and diseases. *FEBS J.* (2016) 283:1607–30. 10.1111/febs.13614 26645578

[12] Wang M Liu J Zhao Y He R Xu X Guo X Upregulation of METTL14 mediates the elevation of PERP mRNA N^6^ adenosine methylation promoting the growth and metastasis of pancreatic cancer. *Mol Cancer.* (2020) 19:130. 10.1186/s12943-020-01249-8 32843065 PMC7446161

[13] Dorn LE Lasman L Chen J Xu X Hund TJ Medvedovic M The N^6^-methyladenosine mRNA methylase METTL3 controls cardiac homeostasis and hypertrophy. *Circulation.* (2019) 139:533–45. 10.1161/CIRCULATIONAHA.118.036146 30586742 PMC6340720

[14] Chen Y Peng C Chen J Chen D Yang B He B WTAP facilitates progression of hepatocellular carcinoma via m6A-HuR-dependent epigenetic silencing of ETS1. *Mol Cancer.* (2019) 18:127. 10.1186/s12943-019-1053-8 31438961 PMC6704583

[15] Jiang ZX Wang YN Li ZY Dai ZH He Y Chu K The m6A mRNA demethylase FTO in granulosa cells retards FOS-dependent ovarian aging. *Cell Death Dis.* (2021) 12:744. 10.1038/s41419-021-04016-9 PMC8316443 34315853

[16] Qiu X Yang S Wang S Wu J Zheng B Wang K M ^6^ A demethylase ALKBH5 regulates PD-L1 expression and tumor immunoenvironment in intrahepatic cholangiocarcinoma. *Cancer Res.* (2021) 81:4778–93. 10.1158/0008-5472.CAN-21-0468 34301762

[17] Mapperley C van de Lagemaat LN Lawson H Tavosanis A Paris J Campos J The mRNA m6A reader YTHDF2 suppresses proinflammatory pathways and sustains hematopoietic stem cell function. *J Exp Med.* (2021) 218:e20200829. 10.1084/jem.20200829 33156926 PMC7653684

[18] Pan Y Ma P Liu Y Li W Shu Y. Multiple functions of m6A RNA methylation in cancer. *J Hematol Oncol.* (2018) 11:48. 10.1186/s13045-018-0590-8 PMC5870302 29587823

[19] Du Y Ma Y Zhu Q Liu T Jiao Y Yuan P An m6A-related prognostic biomarker associated with the hepatocellular carcinoma immune microenvironment. *Front Pharmacol.* (2021) 12:707930. 10.3389/fphar.2021.707930 34248650 PMC8263919

[20] Jiang L Zhang M Wu J Wang S Yang X Yi M Exploring diagnostic m6A regulators in endometriosis. *Aging (Albany NY).* (2020) 12:25916–38. 10.18632/aging.202163 33232273 PMC7803542

[21] Xie Z Yu W Zheng G Li J Cen S Ye G TNF-alpha-mediated m(6)A modification of ELMO1 triggers directional migration of mesenchymal stem cell in ankylosing spondylitis. *Nat Commun.* (2021) 12:5373. 10.1038/s41467-021-25710-4 34508078 PMC8433149

[22] Luo Q Zhang L Li X Fu B Guo Y Huang Z Identification of circular RNA hsa_circ_0044235 and hsa_circ_0068367 as novel biomarkers for systemic lupus erythematosus. *Int J Mol Med.* (2019) 44:1462–72. 10.3892/ijmm.2019.4302 31432107 PMC6713423

[23] Livak KJ Schmittgen TD. Analysis of relative gene expression data using real-time quantitative PCR and the 2(−Delta Delta C(T)) method. *Methods.* (2001) 25:402–8. 10.1006/meth.2001.1262 11846609

[24] Machado P Landewé R Lie E Kvien TK Braun J Baker D Assessment of spondyloarthritis international society: ankylosing spondylitis disease activity score (ASDA S): defining cut-off values for disease activity states and improvement scores. *Ann Rheum Dis.* (2011) 70:47–53. 10.1136/ard.2010.138594 21068095

[25] Garrett S Jenkinson T Kennedy LG Whitelock H Gaisford P Calin A. A new approach to defining disease status in ankylosing spondylitis: the bath ankylosing spondylitis disease activity index. *J Rheumatol.* (1994) 21:2286–91. 7699630

[26] Lu N Li X Yu J Li Y Wang C Zhang L Curcumin attenuates lipopolysaccharide-induced hepatic lipid metabolism disorder by modification of m6A RNA methylation in piglets. *Lipids.* (2018) 53:53–63. 10.1002/lipd.12023 29488640

[27] Rubio R Depledge D Bianco C Thompson L Mohr I. RNA m ^6^ A modification enzymes shape innate responses to DNA by regulating interferon β. *Genes Dev.* (2018) 32:1472–84. 30463905 10.1101/gad.319475.118 PMC6295168

[28] Yang Y Shen F Huang W Qin S Huang J Sergi C Glucose is involved in the dynamic regulation of m6A in patients with type 2 diabetes. *J Clin Endocrinol Metab.* (2019) 104:665–73. 10.1210/jc.2018-00619 30137347

[29] Luo Q Fu B Zhang L Guo Y Huang Z Li J. Decreased peripheral blood ALKBH5 correlates with markers of autoimmune response in systemic lupus erythematosus. *Dis Markers.* (2020) 2020:8193895. 10.1155/2020/8193895 32685056 PMC7334764

[30] Panneerdoss S Eedunuri VK Yadav P Timilsina S Rajamanickam S Viswanadhapalli S Cross-talk among writers, readers, and erasers of m6A regulates cancer growth and progression. *Sci Adv.* (2018) 4:eaar8263. 10.1126/sciadv.aar8263 30306128 PMC6170038

[31] Luo Q Rao J Zhang L Fu B Guo Y Huang Z The study of METTL14, ALKBH5, and YTHDF2 in peripheral blood mononuclear cells from systemic lupus erythematosus. *Mol Genet Genomic Med.* (2020) 8:e1298. 10.1002/mgg3.1298 32583611 PMC7507441

[32] Xue G Gan X Wu Z Xie D Xiong Y Hua L Novel serological biomarkers for inflammation in predicting disease severity in patients with COVID-19. *Int Immunopharmacol.* (2020) 89:107065. 10.1016/j.intimp.2020.107065 33045571 PMC7532789

[33] Tang G Tong S Yuan X Lin Q Luo Y Song H Using routine laboratory markers and immunological indicators for predicting *Pneumocystis jiroveci* pneumonia in immunocompromised patients. *Front Immunol.* (2021) 12:652383. 10.3389/fimmu.2021.652383 33912176 PMC8071988

[34] Liang T Chen J Xu G Zhang Z Xue J Zeng H Platelet-to-lymphocyte ratio as an independent factor was associated with the severity of ankylosing spondylitis. *Front Immunol.* (2021) 12:760214. 10.3389/fimmu.2021.760214 34804047 PMC8602832

[35] Wang J Su J Yuan Y Jin X Shen B Lu G. The role of lymphocyte-monocyte ratio on axial spondyloarthritis diagnosis and sacroiliitis staging. *BMC Musculoskelet Disord.* (2021) 22:86. 10.1186/s12891-021-03973-8 33453722 PMC7811735

[36] Wu J Yan L Chai K. Systemic immune-inflammation index is associated with disease activity in patients with ankylosing spondylitis. *J Clin Lab Anal.* (2021) 35:e23964. 10.1002/jcla.23964 34418163 PMC8418483

[37] Luo Q Gao Y Zhang L Rao J Guo Y Huang Z Decreased ALKBH5, FTO, and YTHDF2 in peripheral blood are as risk factors for rheumatoid arthritis. *Biomed Res Int.* (2020) 2020:5735279. 10.1155/2020/5735279 32884942 PMC7455827

